# Community-driven tree planting greens the neighbouring landscape

**DOI:** 10.1038/s41598-021-96973-6

**Published:** 2021-09-14

**Authors:** Joshua Buxton, Tom Powell, John Ambler, Chris Boulton, Arwen Nicholson, Rudy Arthur, Kirsten Lees, Hywel Williams, Timothy M. Lenton

**Affiliations:** 1 Global Systems Institute, University of Exeter, Exeter, UK; 2 The International Small Group and Tree Planting Program, Vinalhaven, USA; 3 Institute for Data Science and Artificial Intelligence, University of Exeter, Exeter, UK

**Keywords:** Climate-change mitigation, Climate-change mitigation

## Abstract

Nature-based solutions to climate change are growing policy priorities yet remain hard to quantify. Here we use remote sensing to quantify direct and indirect benefits from community-led agroforestry by The International Small group and Tree planting program (TIST) in Kenya. Since 2005, TIST-Kenya has incentivised smallholder farmers to plant trees for agricultural benefit and to sequester CO_2_. We use Landsat-7 satellite imagery to examine the effect on the historically deforested landscape around Mount Kenya. We identify positive greening trends in TIST groves during 2000–2019 relative to the wider landscape. These groves cover 27,198 ha, and a further 27,750 ha of neighbouring agricultural land is also positively influenced by TIST. This positive ‘spill-over’ impact of TIST activity occurs at up to 360 m distance. TIST also benefits local forests, e.g. through reducing fuelwood and fodder extraction. Our results show that community-led initiatives can lead to successful landscape-scale regreening on decadal timescales.

## Introduction

At least 29% of land is degraded globally, negatively affecting living conditions for 40% of people^1^. Land degradation can be driven by deforestation, grazing and poor cropland management, demographic and economic trends, as well as climatic trends^2^. It reduces both the resilience of terrestrial systems to climate change, and the capacity of agricultural communities to adapt^3^. Extensive land degradation has occurred in sub-Saharan Africa, and is expected to continue in the future^2^, threatening the livelihoods of smallholder farmers by reducing land productivity, eroding soil and reducing soil fertility^4,5^.

Agroforestry can reverse degradation, improve soil quality and increase the resilience of smallholder farmers^6–8^, however perceptions of costs and risks among farming communities with marginal livelihoods mean that uptake of restorative practices has not been widespread^9^. Agroforestry in degraded African landscapes has potential to sequester significant amounts of carbon^10^, indicating the potential for carbon markets as a funding mechanism for environmental rehabilitation^11^ with potentially powerful benefits for participating communities^12^, but the need to aggregate sequestration across large numbers of small farms has been identified as a significant barrier^10^. As such, successful projects are often localised and limited in scalability, and the impacts of grass-roots actions in rural areas are under-quantified. Local knowledge is essential in enabling context-dependent solutions that prioritise the agency of community-members in decision-making^13,14^, and successful and sustained adoption is more likely when farmers are able to adapt new technology themselves and apply in their local context^9^, as well as carrying socio-economic benefits for participants^8^. One project offering a scalable, farmer-led approach that accesses benefits from carbon markets is The International Small Group and Tree Planting Program (TIST)^11,15^.

TIST is a farmer-led network of over 100,000 smallholder farmers in Tanzania, Uganda, Kenya and India, organised around agroforestry and regenerative farming practices^16^. The TIST programme develops and shares best-practices for tree-planting, sustainable agriculture and gender-balanced leadership opportunities, bringing multiple economic, social and health benefits to its members^17^. TIST provides a strong incentive to plant and maintain trees by training farmers to systematically quantify and aggregate tree-growth data, which is packaged as verified carbon credits for sale on international voluntary markets. Trees are generally grown from locally collected seed, with species choice left entirely to the farmer, and many TIST members or their families establish tree nurseries and sell on seedlings to generate extra income. In this study we focus on the Mt Kenya region of Kenya, which has the highest concentration of TIST members across all four countries. Farmers in this region typically grow a range of crops, most commonly including maize, legumes and tubers^18^, with two cropping seasons determined by the two distinct rainy seasons each year.

TIST-Kenya farmers plant a mix of tree species, with over 160 species reported, including more than 90 indigenous species^17,18^. Species selection is dominated by trees that provide the most reliable products including fuelwood, animal fodder and coppiced timber^8^, often supplemented with fruit and nut trees which can provide high value crops. Early in the programme farmers planted a high proportion of *Eucalyptus grandis* due to encouragement by the Kenyan Forest Service (KFS). TIST have since discouraged planting of Eucalyptus, but standing trees from this period still represent a high proportion of the total (up to 33.1% in some project areas^17^). *Grevillea robusta*, a multi-use non-native tree planting of which has been encouraged by World Agroforestry (ICRAF), is also a dominant species, with native and non-native *Acacia* species and a range of others also present in significant numbers.

TIST credits have high value due to their associated social and environmental benefits^17,19^. In addition to the income derived from carbon credit sales, TIST farmers gain substantial benefits via production of fuelwood, animal fodder, food and diversification of livelihoods, with an estimated average value of USD $1,324 per member per year^17,19^. Other benefits include shade provision, reduced soil erosion and increased water penetration^6,20^, with certain TIST activities aimed at maximising these, e.g. targeted planting in riparian areas^17^. Alongside tree-planting, TIST farmers often use sustainable farming practices such as ‘conservation farming’^17,19^, which can improve soil organic matter and water retention through a combination of no-till planting, mulching and cover cropping^21^.

**Figure 1:**
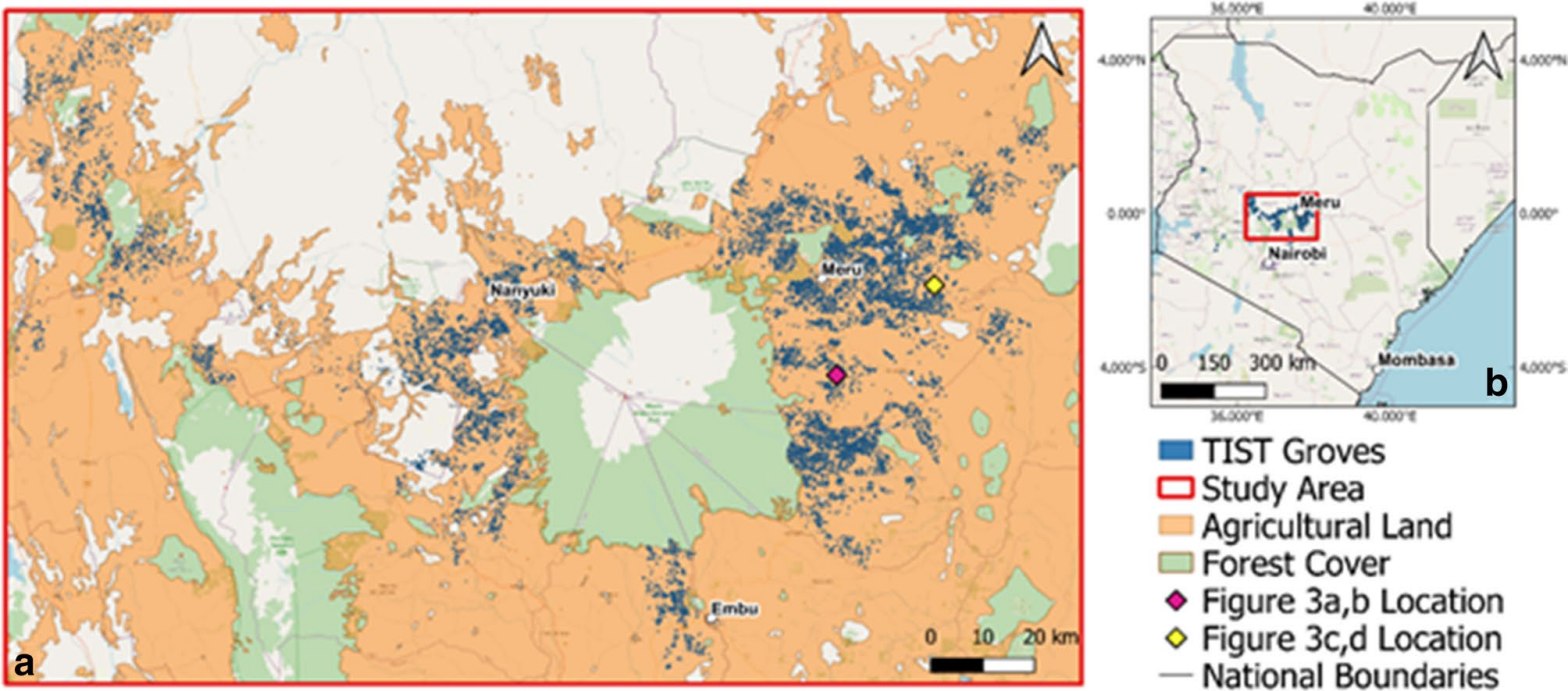
Study area: **(a) **Map of TIST groves in the Mount Kenya region and agricultural land within the study area. TIST groves are clustered and spatially correlated within the study area due to the spread of TIST through community networks. **(b)** Map of TIST groves and study area within Kenya. Map data: © OpenStreetMap contributors. Figure created with QGIS.

Here we use Landsat 7 satellite data to examine whether TIST farmers in Kenya have achieved regreening within their own farms and at landscape scale. Figure 1 shows the location of TIST tree-groves in Kenya and the study region. We analyse trends in the Normalised Difference Vegetation Index (NDVI), a measure of plant greenness^22^, over the period 2000–2019 at 34,699 TIST tree-groves established since 2005. A ‘grove’ represents a defined tree planting area within the boundaries of a farm, which we compare with the wider agricultural landscape. Trends in NDVI are measured using Mann–Kendall’s Tau rank correlation coefficient; this statistic indicates the tendency of a trend, with τ = 1 showing a continuous increase, τ = 0 showing no trend, and τ = − 1 showing a continuous decrease. Therefore pixels with a τ > 0 value display a ‘greening’ trend, while those with τ < 0 display a ‘browning’ trend. As detailed below, we further classify these greening and browning trends as ‘moderate’ or ‘strong’, following Gichenje and Godinho^23^. Other studies have used similar methods to analyse trends in NDVI and land degradation across Kenya with lower resolution data^23,24^.

## Results

**Figure 2:**
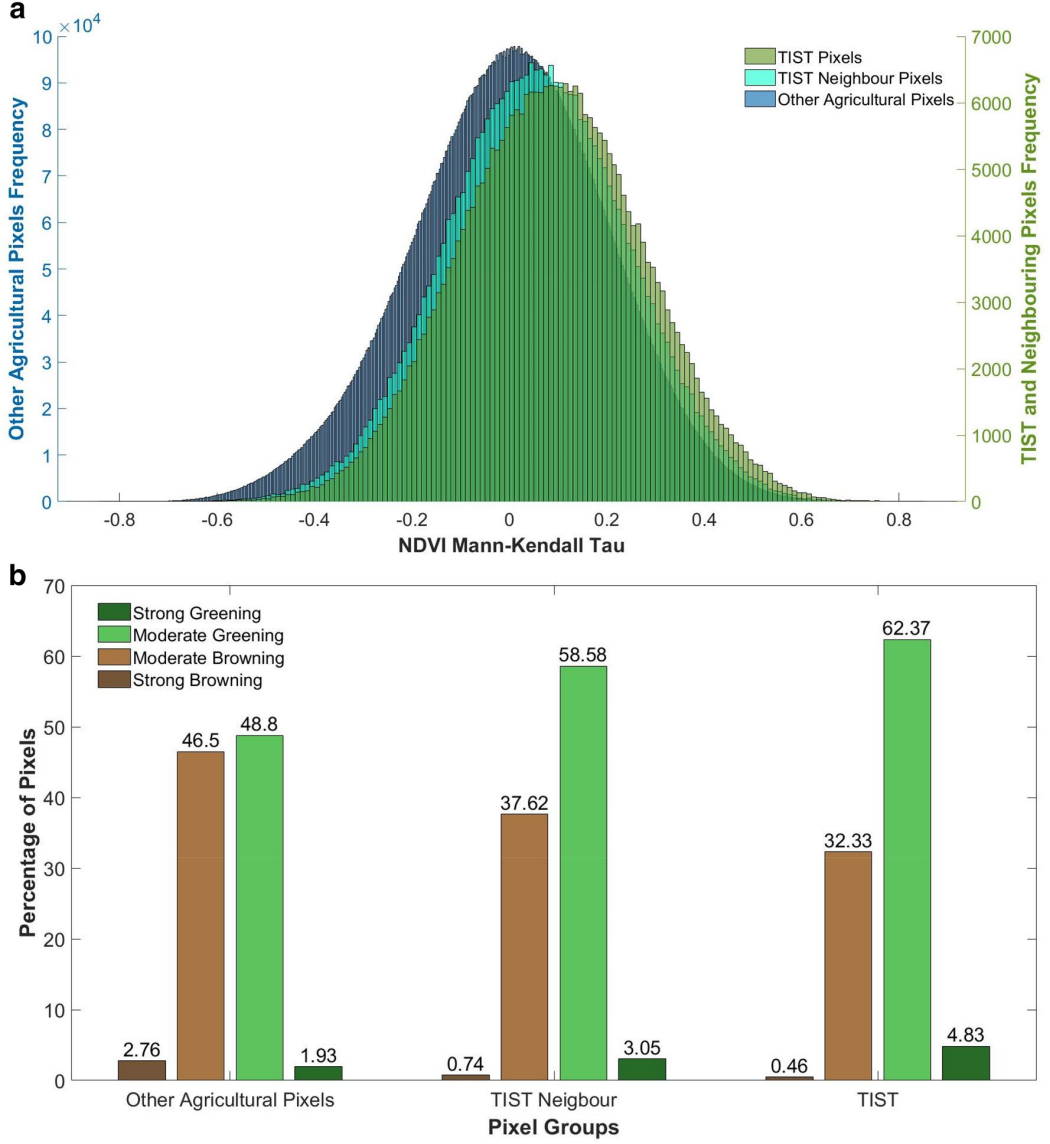
Greening trends across study area: **(a)** distribution of NDVI Kendall Tau for TIST groves, neighbouring pixels and other agricultural pixels within the study area. Trends in NDVI Kendall Tau are positively shifted for TIST groves and neighbouring pixels relative to other agricultural pixels within the study area. **(b)** Classified greening and browning trends within TIST groves, TIST Neighbour pixels and other agricultural pixels. Based upon Mann–Kendall Tau values pixels are binned into ordered groups showing “strong browning” (−0.8 < τ < −0.4), “moderate browning” (−0.4 < τ < 0), “moderate greening” (0 < τ < 0.4) and “strong greening” (0.4 < τ < 0.8), following Gichenje and Godinho^23^. TIST grove pixels and TIST neighbouring pixels show a larger proportion of greening pixels than other agricultural land. Over 67% of TIST grove pixels show some form of greening compared to 51% of other agricultural land.

Figure 2a shows the distribution of NDVI Mann–Kendall Tau values for pixels which subdivide the study area into three landscape classes: TIST groves, neighbouring pixels and other agricultural pixels. Due to the 30 m resolution of Landsat, TIST groves are defined as those which have a centroid either within a TIST grove or a 15 m radius from a grove boundary, while neighbouring pixels are defined such that they fall within a 30 m buffer of TIST pixels. These neighbouring pixels represent farmland upon which TIST farmers may utilise sustainable agricultural practices, as well as neighbouring farms.

As can be seen in Fig. 2b, the majority (67.20%) of TIST grove pixels and neighbouring pixels (61.63%) display a positive greening trend, while other agricultural pixels display a broadly neutral trend. A two sampled t-test determined that TIST groves are significantly different to other agricultural areas (p < 0.001). This suggests that TIST groves are distinct from the wider landscape and that trees planted by farmers contribute an observable change. While not all TIST grove pixels display an absolute greening trend, it is worth noting that there is a large amount of variability across the TIST network in the number and density of trees planted in a grove. Some groves consist of boundary trees or windbreaks, with crops, buildings or other land use types included within the grove, these may cause a browning trend. Other factors which may cause browning within TIST groves are climatic trends, the possibility that the groves are unsuccessful or the farmer leaves TIST. Neighbouring pixels display a closer similarity to TIST groves than to the rest of the landscape and appear to have a stronger greening trend than other agricultural pixels. TIST neighbouring pixels are statistically significantly different to other agricultural regions (p < 0.001), as well as to TIST groves (p < 0.001).

**Figure 3:**
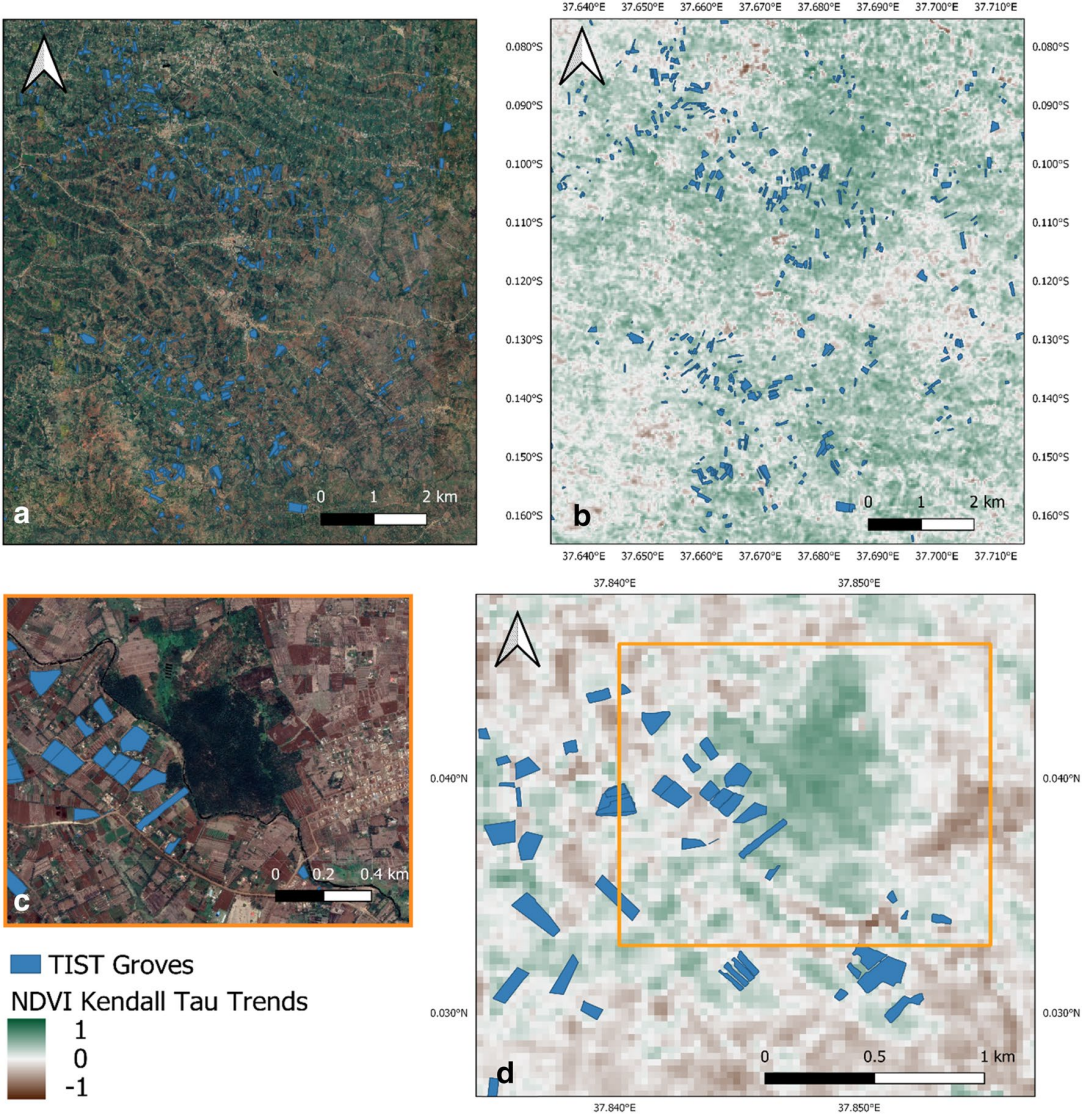
Examples of greening in the vicinity of TIST groves: **(a,b)** Example of a group of TIST sites within a greening agricultural landscape. **(a)** TIST sites within a heterogenous landscape. **(b)** NDVI Kendall Tau greening trends for this area. **(c,d)** TIST sites in Meru County bordering a small woodland. **(c)** Extent of this small woodland area neighbouring TIST groves. **(d)** Greening of the woodland. This greening effect is likely to be caused by a reduction in the extraction of firewood and forage from these woodlands and represents a very specific form of natural vegetation improvement. **(a,c) **Map data: ©2020 Google, CNES/Airbus, Maxar Technologies. Figure created with QGIS.

Spill-over effects in the surrounding agricultural landscape due to TIST farmers are likely to be the result of several factors, such as the sustainable agriculture employed by these farmers, as well as the microclimatic effects of tree planting. Figure 3a,b shows an example of a group of TIST groves which are situated within a wider greening of agricultural land.

Discussions with TIST farmers suggest that in areas with numerous TIST groves there can be co-benefits to local woodland as well as agricultural land. Figure 3c,d shows an example of this, with numerous TIST groves bordering a confirmed natural woodland site which has a positive NDVI Kendall Tau. This strong positive trend is noticeable within the borders of these woodland areas and is in addition to improvements in farmland due to the restorative activities of TIST farmers. This woodland greening trend serves as an additional environmental benefit to the positive trends seen in agricultural land adjacent to TIST sites. Woodland improvements are attributed to a reduction in human pressures on woodland for firewood and forage for animals. Other reasons given by farmers is the creation of woodland protection and education groups due to the increased environmental awareness promoted by TIST membership. TIST have also encouraged members to establish Community Forest Associations (CFAs)^17^.

**Figure 4:**
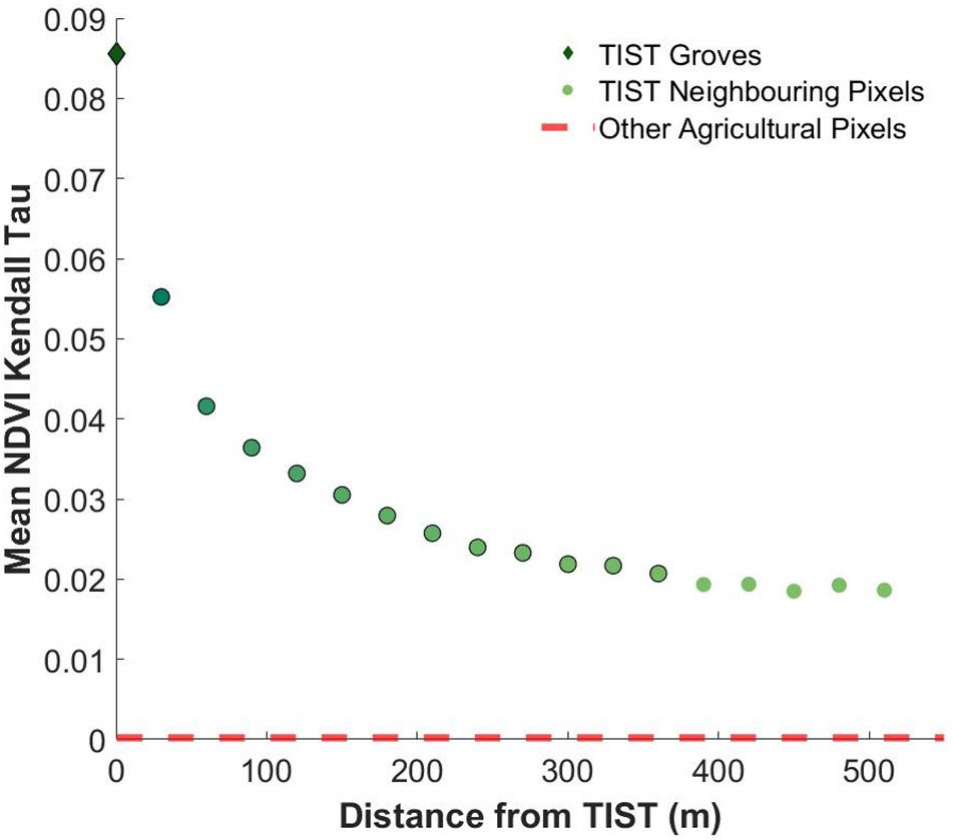
Average NDVI Kendall Tau of TIST groves compared with neighbouring pixels at increasing distances: Greening effect of TIST is observable in neighbouring pixels and then declines with distance from TIST groves. The red line represents the average NDVI Kendall Tau value for all Non TIST values in the study area. Distances which display a significant TIST effect are represented with a black outline. Standard error not shown due to very small size, provided in the Supplementary Table 1.

Figure 4 shows the NDVI trend at increasing distance from TIST sites. TIST groves’ immediate neighbours have a stronger average greening trend than those which are further away. This TIST effect seems to decay with distance, until it reaches a local background greening level which is higher than the average trend across the agricultural land within the study area. This suggests that TIST groves are clustered in areas that already show a weak greening trend, but that TIST activities contribute to a greening trend of greater magnitude on top of this. TIST groves are themselves spatially correlated to a degree, as can be seen in Fig. 1, due to the way that TIST spreads through local community networks. While TIST groves tend to be in areas that experience a greening trend (Supplementary Fig. 1), background greening is not exclusive to areas with a high density of TIST groves.

The benefits of TIST, such as microclimatic effects and the use of sustainable farming practices adjacent to tree-groves, decline with distance hence the decay in greening. An analysis of the TIST effect over distance considers the asymptotic nature of the curve in Fig. 4. With the use of a categorical regression model, as detailed in the methodology, we establish that pixels do not reach the local background level of greening until 390 m away from TIST groves (Supplementary Table 2). This suggests a secondary TIST effect present up to 360 m away, although this weakens greatly with distance.

## Discussion

Here we have shown that the activities of a community-led tree planting programme are observable using Landsat 7 satellite imagery as increasing greening trends that are clearly distinguishable from the wider landscape. TIST groves display a trend not seen in the rest of the study area, with over 67% of TIST pixels displaying a greening effect, compared to 51% of other agricultural land in the study area. This greening is directly associated with tree planting by farmers in TIST tree-groves over a total area of 27,198 ha in our study area.

**Figure 5:**
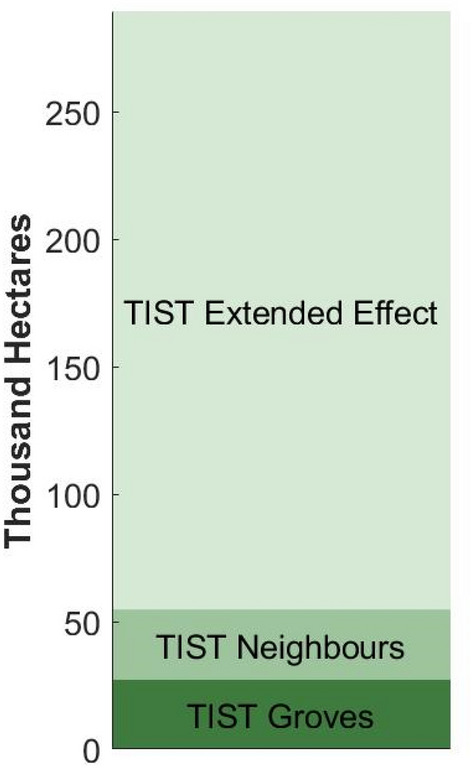
Total extent of greening associated with TIST groves.

In addition to this, we observe an overspill effect, with land near to TIST groves showing a greening trend. This effect is most strongly observed in pixels immediately adjacent to tree groves, suggesting direct impacts of TIST activities on an additional 27,750 ha of farmland; an area slightly greater than that of the groves themselves. Weaker, but statistically significant, effects can be observed up to 360 m away from TIST groves, suggesting that the full extent of TIST impacts in the study area may extend across a further 234,720 ha, leading to a total area of 289,668 ha (Fig. 5). This spill-over effect highlights a substantive benefit of mosaic tree-planting schemes and shows that membership of TIST, which promotes both tree planting and sustainable agriculture, can have landscape level impacts.

Spill-over effects from agroforestry programs have previously been identified with regards to community capital and knowledge^25^, and ’positive leakage’ of carbon sequestration^26^. Spill-over impacts on NDVI trends in our study area are likely to be caused by multiple interacting drivers. Immediately in and around TIST groves, reduced soil erosion, increased soil nutrition and increased shade from TIST planted trees is likely to benefit other vegetation^6,20^. There is also evidence that managed tree planting within dry tropical areas can improve groundwater recharge^27^, which is likely to be of benefit in some TIST groves. In addition to tree-planting, TIST is an example of community capital that serves an important role in generating and spreading other best practices for sustainable land management. Higher yields and the use of cover crops associated with ’conservation farming’^21^ are likely to play a significant role in the strong greening trend in pixels immediately adjacent to TIST groves, many of which will fall within the boundaries of farms belonging to TIST members. Furthermore, as TIST membership itself is most commonly spread via neighbour-to-neighbour interactions, adoption of beneficial farming practices likely spreads in the same way.

A secondary effect identified by TIST farmers suggests that areas of natural woodland can be improved by the presence of TIST farms, as shown in Fig. 3c. As farmers produce more animal fodder and firewood within their farm^17^, they are less likely to remove vegetation from local woodland, thus reducing anthropogenic pressure on the woodland^8,28,29^. In addition, farmers have become more environmentally aware due to their engagement with TIST^30^ and some have joined with forest protection groups to educate others on the dangers of depleting local woodlands^31^. Further research is needed to understand the full extent of these effects.

There is currently little available literature which combines remote sensing with grassroots community tree planting. Much of the focus is placed upon large scale, government led projects, such as that in semi-arid Northern China^32^. These tree planting projects are often driven from the top down and occur over a large contiguous area, in contrast to farmer-led initiatives that contribute to development of rural livelihoods and which are likely to have more successful adoption and more sustained impacts^9,14^. Governments worldwide have announced tree planting projects with large target numbers^33^, with international efforts such as the Trillion Tree Campaign and Africa’s Great Green Wall. Our results show that the cumulative impacts of a bottom-up, community-driven tree planting initiative can contribute to rehabilitating degraded landscapes, while enabling local farmers to access direct economic benefits from carbon credit payments, substantial indirect benefits from diversified livelihoods and farm improvements^11,17,19^ and agricultural, health and other training opportunities^17^. These restoration schemes can have multiplier effects, with improvements occurring in land adjacent to tree planting sites. This restoration can be achieved with the engagement of local people, with organisations like TIST providing agency to farmers and highlighting the value that these grassroots initiatives can have. We suggest that enhanced community capital and the biophysical and agro-ecological impacts of TIST best-practices combine in a reinforcing feedback to produce widespread, landscape-scale effects, with such programmes representing a mechanism to initiate rapid positive change^34^.

## Methodology

### Study area

The study area outlined in Fig. 1 covers a total area of 26,864 km^2^ and was chosen to include TIST groves in proximity to Mount Kenya and the Aberdare Mountain Range. This area constitutes the majority of TIST groves within Kenya as seen in Fig. 1. The expansion and intensification of agricultural land in the region of Mount Kenya has occurred at the expense of the natural environment, with increasing pressure associated with a growing population^35^. Within the Mount Kenya region there exists two rainy and two dry seasons per year. The topography of the region means that there is much spatial variability in rainfall, with mean annual rainfall varying between 600 mm year^−1^ and > 1300 mm year^36^.

As well as the bounding box around this region, satellite data was then filtered to only include agricultural areas as defined by the FAO^37^. This agricultural boundary ensures the removal of pixels within National Parks and forests, large urban areas and other non-agricultural regions, the extent of this is shown in Fig. 1.

### Data

Grove locations and shapefiles were supplied by TIST for this analysis. These shapefiles are constructed of the perimeter of TIST groves and are recorded by TIST quantifiers who walk the boundary of the grove several times to get an accurate measurement using GPS trackers. These groves are usually distinct areas of farms where TIST members have chosen to plant trees and often represent degraded land^17^ where other crops may not be suitable. However, it is not uncommon for farmers to plant trees around the borders of their farm, meaning that some grove shapefiles will contain the whole farm. TIST grove data in Kenya is provided from the beginning of 2005 up to the end of 2018 and includes TIST groves which have been validated. The median grove size in the study area is 0.285 ha, with the median number of trees being 83.

Due to the establishment of TIST in Kenya in 2005 and the small size of TIST groves, it was necessary to select a satellite which had both sufficient temporal coverage and spatial resolution. Landsat 7 EMT + data was used because of its 30 m resolution and availability since 1999^38^. The study period was from January 2000 to December 2019, as this is the largest full year extent available for the satellite. The Landsat 7 Collection 1 Tier 1 8-Day NDVI Composite was selected from the GEE data repository.

This 8 day NDVI composite dataset was used to calculate monthly data based upon the monthly maximum value composite technique. This method selects the maximum value for each pixel within a monthly period and is used in order to reduce the impact of water vapour, cloud cover, aerosols and the angle of the sun^39^. This monthly NDVI data was then used to calculate multi-annual monthly averages for the period.

### Analysis

The trend in NDVI was calculated using the following steps (see Supplementary Fig. 2 for workflow). To remove the seasonal cycle from the data, the multi-annual monthly averages are subtracted from the monthly maximum composite. This creates a decycled dataset. Then, to smooth and detrend the data, a 12-month moving average is taken of the decycled data. Any values which did not fall within a full 12 month window are then removed.

The non-parametric Mann–Kendall Tau, or Kendall’s Tau, is a common test of the trend of a time series. It provides a value between − 1 and 1. The Kendall Tau of the detrended and decycled NDVI data set is calculated, with Kendall Tau values of greater than 0 suggesting a greening trend, and thus more vegetation, with values less than 0 suggesting a browning trend of a pixel, and therefore less vegetation.

These pixels are then separated into TIST pixels, TIST neighbour pixels and other agricultural pixels. A pixel is classed as a TIST pixel if its centroid falls within a 15 m buffer of a TIST grove. This is to ensure that border areas of TIST groves are included as these are often where a farmer will plant their trees. Pixels are classed as neighbouring to a TIST grove if their centroid falls within a 30 m buffer of a TIST pixel. This 30 m buffer represents the size of a Landsat pixel. Other agricultural pixels are defined as those which are neither TIST or neighbouring pixels and fall within the agricultural area as defined by the FAO^37^and within the study area.

This method of calculating neighbours is then applied to each successive set of neighbours, with the buffer increasing by 30 m each time. Each distance class only includes unique values and ignores values that might be within a smaller distance class. This is done to calculate any spill-over effects of TIST groves, as well as to compare the groves to their local trend. We include all pixels within the study area for the neighbouring analysis to consider non-agricultural land such as national parks.

The magnitude of the trends in greening and browning are then classified as strong browning (− 0.8 < τ < − 0.4), Moderate Browning (− 0.4 < τ < 0), Moderate Greening (0 < τ < 0.4) and Strong Greening (0.4 < τ < 0.8)^23^. Two sample t-tests were used to assess for statistically significant differences between TIST pixels, neighbouring pixels and other agricultural areas.

To assess the effect of TIST upon neighbouring agricultural land, as shown in Fig. 4, we considered the declining effect with distance as an asymptotic curve. We consider the largest three distances, at 450 m, 480 m and 510 m, as an asymptote of this curve and take the mean of these values. We then fit a categorical regression model with an intercept of this mean and with categories corresponding to each distance class. We then test whether coefficient of each category is statistically different to the intercept (the asymptote value). If a coefficient is statistically different then this suggests that the TIST effect is present, while if it is not statistically different then that distance class does not differ from the local background level.

The remote sensing component of this study was undertaken primarily with Google Earth Engine (GEE)^40^. This data was then extracted from GEE for further analysis in QGIS^41^, Matlab R2020a and R^42^.

### Limitations

There are well known difficulties with cloud cover when using remote sensed data to conduct time series analysis. These difficulties are also compounded by the well documented scan line error of the Landsat 7 satellite^38^. To create a continuous time series and to reduce the influence of cloud cover, this study has relied on decycling and detrending techniques. A reduction in cloud cover influence was also achieved by the use of maximum monthly pixel composites^39^. An additional limitation is presented by the TIST grove data. As some TIST groves include the entirety of a farm, this will include cropland and buildings. These are likely to influence the trends in NDVI.

## Supplementary Information

Supplementary Information.
