# Supplementary material for: Community-driven tree planting greens the neighbouring landscape
Source: Sci Rep. 2021 Sep 14;11:18239. doi: 10.1038/s41598-021-96973-6 (PMC8440767; doi:10.1038/s41598-021-96973-6)
Supplement: Supplementary file 1 — Supplementary Information. [file 41598_2021_96973_MOESM1_ESM.docx]

Community-driven tree planting greens the neighbouring landscape – **Supplementary Information**

Joshua Buxton, Tom Powell, John Ambler, Chris Boulton, Arwen Nicholson, Rudy Arthur, Kirsten Lees, Hywel Williams, Timothy M. Lenton

**
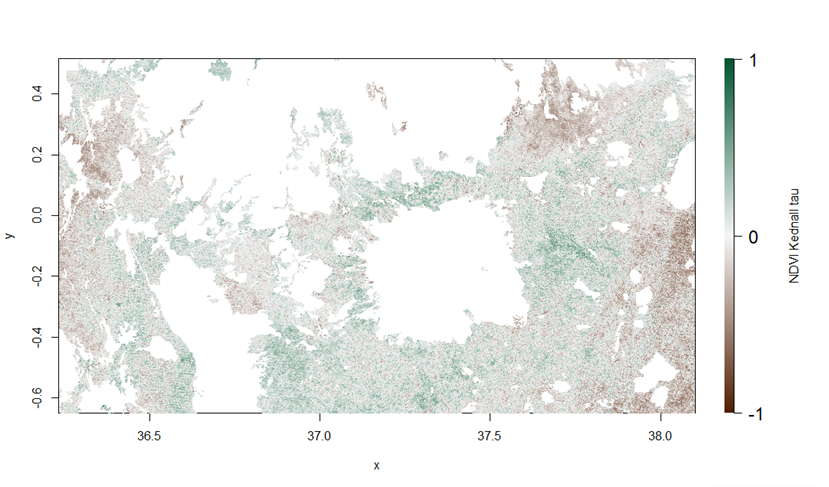
**

**Supplementary Figure 1: Map of NDVI Kendall Tau trends across the agricultural land within the study area for the period 2000-2019.**

Figure created in R.


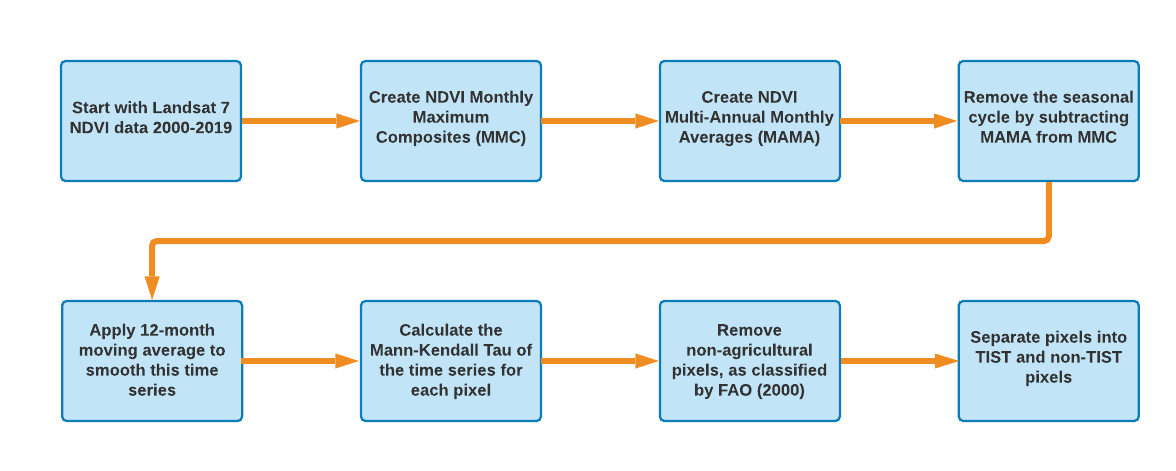


**Supplementary Figure 2: Data analysis workflow for calculation of NDVI Kendall Tau trends across the study area.Supplementary Table 1: Standard error of the mean for NDVI Kendall Tau values of TIST neighbouring classes.**

| Distance from TIST (m) | Standard Error of the Mean |
| --- | --- |
| 0 | 3.43E-04 |
| 30 | 3.34E-04 |
| 60 | 3.32E-04 |
| 90 | 3.38E-04 |
| 120 | 3.49E-04 |
| 150 | 3.62E-04 |
| 180 | 3.74E-04 |
| 210 | 3.88E-04 |
| 240 | 4.09E-04 |
| 270 | 4.15E-04 |
| 300 | 4.30E-04 |
| 330 | 3.44E-05 |
| 360 | 3.45E-05 |
| 390 | 3.45E-05 |
| 420 | 3.46E-05 |
| 450 | 3.48E-05 |
| 480 | 3.48E-05 |
| 510 | 3.49E-05 |

**Supplementary Table 2: Values from the Categorical Regression Model used to assess the extent of TIST’s landscape effects.** Values which are significant with a p-value <0.05 are presented in bold. This suggests that areas up to and including 360 metres display a distinct difference to the local background greening trend.

| Distance  (metres) | Estimate | Std. Error | p-value |
| --- | --- | --- | --- |
| **0** | **0.0668** | **0.000342** | **<2e-16** |
| **30** | **0.0364** | **0.000339** | **<2e-16** |
| **60** | **0.0228** | **0.000335** | **<2e-16** |
| **90** | **0.0176** | **0.000341** | **<2e-16** |
| **120** | **0.0144** | **0.00035** | **<2e-16** |
| **150** | **0.0117** | **0.000363** | **<2e-16** |
| **180** | **0.00914** | **0.000375** | **<2e-16** |
| **210** | **0.00694** | **0.000389** | **<2e-16** |
| **240** | **0.00518** | **0.000409** | **<2e-16** |
| **270** | **0.00449** | **0.000416** | **<2e-16** |
| **300** | **0.00308** | **0.00043** | **6.85E-13** |
| **330** | **0.0029** | **0.000446** | **8.09E-11** |
| **360** | **0.00191** | **0.000456** | **2.75E-05** |
| 390 | 0.000541 | 0.000468 | 0.248 |
| 420 | 0.000582 | 0.000486 | 0.231 |
| 450 | -0.0003 | 0.000492 | 0.548 |
| 480 | 0.00046 | 0.000501 | 0.358 |
| 510 | -0.00016 | 0.00051 | 0.754 |
